# A Programmable Crypto-Processor for National Institute of Standards and Technology Post-Quantum Cryptography Standardization Based on the RISC-V Architecture

**DOI:** 10.3390/s23239408

**Published:** 2023-11-25

**Authors:** Jihye Lee, Whijin Kim, Ji-Hoon Kim

**Affiliations:** Department of Electronic and Electrical Engineering, Ewha Womans University, Seoul 04763, Republic of Korea; jihyelee317@gmail.com (J.L.); whijin98@gmail.com (W.K.)

**Keywords:** post-quantum cryptography, PQC, RISC-V, crypto-processor, programmability, domain-specific processor

## Abstract

The advancement of quantum computing threatens the security of conventional public-key cryptosystems. Post-quantum cryptography (PQC) was introduced to ensure data confidentiality in communication channels, and various algorithms are being developed. The National Institute of Standards and Technology (NIST) has initiated PQC standardization, and the selected algorithms for standardization and round 4 candidates were announced in 2022. Due to the large memory footprint and highly repetitive operations, there have been numerous attempts to accelerate PQC on both hardware and software. This paper introduces the RISC-V instruction set extension for NIST PQC standard algorithms and round 4 candidates. The proposed programmable crypto-processor can support a wide range of PQC algorithms with the extended RISC-V instruction set and demonstrates significant reductions in code size, the number of executed instructions, and execution cycle counts of target operations in PQC algorithms of up to 79%, 92%, and 87%, respectively, compared to RV64IM with optimization level 3 (-O3) in the GNU toolchain.

## 1. Introduction

With the emergence of 5G communication and the development of various services, including Internet-of-Things and cloud services, a large amount of personal information, such as medical and financial records, is transmitted through communication channels. Public-key cryptography has been commonly used as a traditional method for maintaining confidentiality and integrity and preventing attackers or sniffers on the channel from disclosing this information. Diffie and Hellman introduced public-key cryptography in 1976 [1]. The cryptosystem is based on a complex mathematical problem that takes a long time to solve. Traditional public-key cryptography schemes, such as Elliptic-Curve Cryptography (ECC) [2] and the Rivest–Shamir–Adleman (RSA) algorithm [3], are based on the discrete logarithm problem and factorization problem, respectively.

In 1994, Peter Shor introduced Shor’s algorithm [4], which proves that quantum computers can solve the mathematical problems for ECC and the RSA algorithm in polynomial time. This discovery makes quantum computers a potential threat to the security of traditional public-key cryptography. In recent years, Google has proposed a quantum processor called Sycamore, which takes about 200 s to solve an operation that would take a state-of-the-art supercomputer about 10,000 years to complete. In addition, IBM Q System One was introduced in 2019, and related studies are underway. As quantum computers are now widely deployed worldwide, it is necessary to develop quantum-resistant cryptosystems to ensure information security; this is known as post-quantum cryptography (PQC). As the importance of quantum-safe algorithms is well understood globally [5] and various cryptography algorithms have been developed, the National Institute of Standards and Technology (NIST) initiated the standardization process of post-quantum cryptography in 2017.

**Figure 1:**
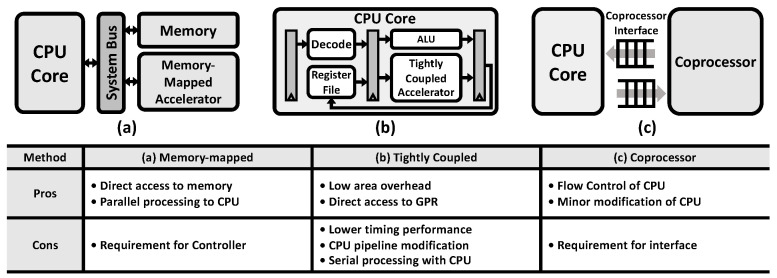
Comparison of hardware accelerator implementation methods. (**a**) Memory-mapped accelerator, (**b**) tightly coupled accelerator, and (**c**) coprocessor.

The number of PQC publications in the past 5 years has accounted for nearly half of the articles published in this field over the past 25 years [6]. Previous works have studied the implementation of post-quantum cryptography algorithms [7]. However, due to various schemes, frequent memory access, and iterative operations, it is inefficient to implement these algorithms solely in software [8]. Various studies on hardware accelerators have recently been proposed and can be categorized into three types of implementation methods, as shown in Figure 1 [9].

The first method attaches a memory-mapped accelerator to system buses such as AMBA AXI and AHB. The CPU core accesses this accelerator by storing values in a specific memory space allocated to the accelerator, which triggers a specific operation depending on the data written to the memory address [10,11]. This method does not require modifications to the CPU core’s microarchitecture and enables parallel processing alongside the CPU. However, the memory-mapped accelerator leads to additional memory access latency due to load and store operations for workload offloading to the accelerator.

The second method implements a tightly coupled accelerator within the CPU microarchitecture as a functional unit. This method allows easy access to the general-purpose register file in the CPU core and incurs low area overhead. However, custom instructions must be decoded before execution, which requires modifications to the CPU’s microarchitecture. Additionally, concurrent operation with the main thread in the CPU core is limited in a single-thread processor, resulting in lower performance compared to an external accelerator. Previous works, such as [12,13,14], defined custom RISC-V instruction sets for targeted post-quantum cryptography algorithms.

The third method involves a coprocessor attached to the CPU core through a dedicated interface. This method is widely adopted in modern CPU architectures such as Arm and RISC-V and allows for easy extension of the CPU core’s capabilities with additional coprocessor instructions, without significant modifications to the baseline CPU’s microarchitecture [9,15]. A high-performance vector coprocessor for lattice-based cryptography was designed in [16].

Our proposed programmable crypto-processor aligns with the coprocessor scheme, which reduces the design complexity caused by the use of a system bus and memory map, in comparison to studies involving memory-mapped PQC accelerators [10,11]. Also, compared to previous studies [12,13,14], there is no need to change the internal design of the existing CPU core because of the introduction of the coprocessor interface in this paper, which can connect the proposed crypto-processor to the main CPU core.

**Figure 2:**
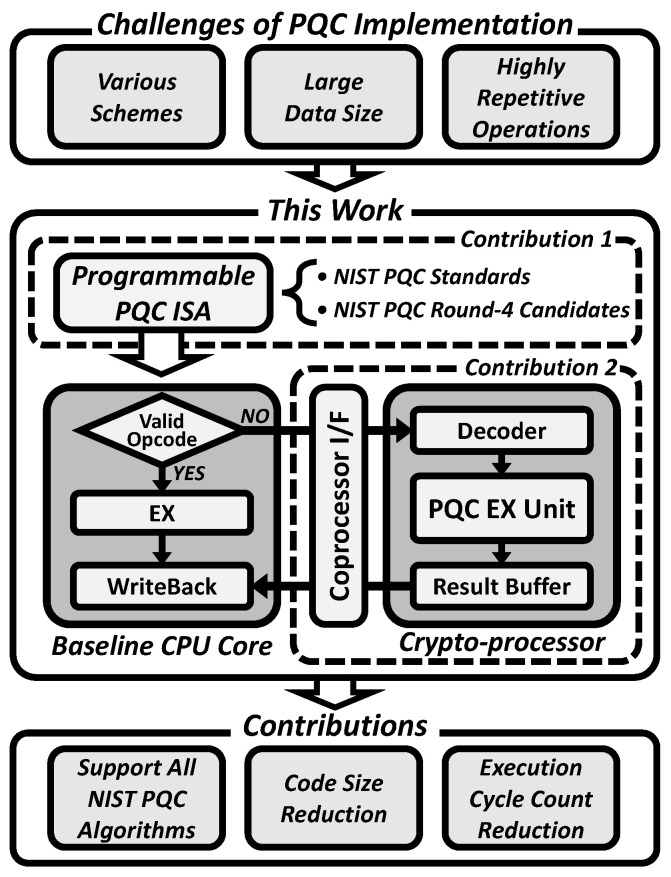
Proposed crypto-processor based on RISC-V architecture.

Several studies have been conducted on accelerating lattice-based cryptography, such as Kyber and Dilithium. Previous works, such as [11,16], mainly focused on accelerating lattice-based algorithms. However, less attention has been given to the hardware acceleration of other cryptographic schemes. Although various PQC schemes based on mathematical bases have been developed, as described in [8,17], hardware implementations of PQC algorithms commonly require a large memory footprint and highly repetitive computations, which are significant obstacles in efficient hardware implementations. To address this issue, this paper proposes a custom RISC-V Instruction Set Architecture (ISA) extension that can support all round 4 candidate algorithms in the NIST PQC standardization process, as well as algorithms yet to be standardized. This paper also introduces a programmable crypto-processor that can be attached to the main RISC-V CPU core via a widely used coprocessor interface, known as the CORE-V extension interface [15]. With the proposed RISC-V instruction set extension, the crypto-processor can flexibly support various PQC algorithms across various applications. Furthermore, this approach can reduce the code size of cryptosystems, leading to a smaller instruction memory requirement for PQC computation. Additionally, the number of executed instructions in the overall architecture and the execution clock cycle are significantly improved compared to baseline RISC-V implementations (Figure 2).

The remainder of this paper is organized as follows. Section 2 provides an overview of the targeted NIST PQC algorithms and their analysis from a performance point of view. Section 3 discusses the proposed instruction set for NIST PQC algorithms, including the algorithms to be standardized, round 4 candidates, and crypto-processor architecture that can support custom instructions. Section 4 presents the experimental results and the discussion, including a comparison with previous works. Finally, Section 5 contains the conclusions drawn in this paper.

## 2. Target PQC Algorithms

PQC algorithms can be categorized based on their arithmetic foundations. This section provides an overview of the NIST PQC standardization algorithms and their arithmetic background. Additionally, we describe their fundamental operations, which can result in performance bottlenecks in hardware implementations.

### 2.1. NIST PQC Standard and Round 4 Algorithms

The target PQC algorithms in this paper are the round 4 candidates of NIST PQC standardization and the algorithms selected to be standardized, as declared on 5 July 2022, as denoted in Table 1. The algorithms can be categorized into public-key encryption/key-establishment or digital signature algorithms.

**Figure 3:**
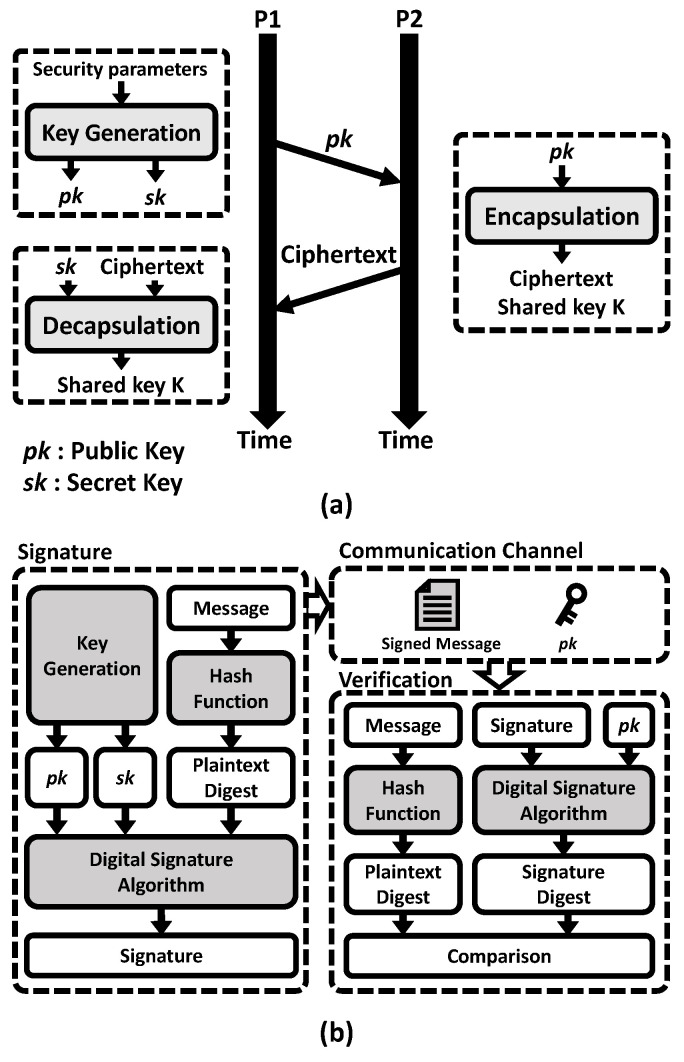
Fundamental flow of (**a**) key-establishment mechanism, (**b**) digital signature algorithm.

Figure 3a depicts the fundamental operation flow of the key-establishment mechanism that enables key establishment. This mechanism comprises three main components: key generation, encapsulation, and decapsulation. During key generation, security parameters are input, generating a pair consisting of a public key (pk) and a secret key (sk). The public key pk is then transmitted to the P2 side. Encapsulation receives pk as input and produces a ciphertext and a shared key *K*. The ciphertext, an encapsulation of the key *K*, is sent to the P1 side. Finally, decapsulation returns a shared key based on a secret key and the ciphertext [18].

The digital signature algorithm consists of key generation, a signature, and verification steps, as shown in Figure 3b. First, the sender generates a public key, pk, and a secret key, sk, for communication. Next, the message is input to the hash function and signed by the secret key. Finally, the receiver verifies the sender’s authenticity by comparing the plaintext digest and the signature digest using the sender’s public key [19].

The PQC algorithms targeted in this paper can be categorized into lattice, hash, and code-based algorithms based on their arithmetic basis, as indicated in Table 1. Specifically, CRYSTALS-Kyber and CRYSTALS-Dilithium are based on the module-LWE problem, whereas SPHINCS+ relies on the security of hash functions such as SHAKE256, SHA-256, and Haraka. Code-based algorithms, including BIKE, HQC, and Classic McEliece, are based on quasi-cyclic moderate-density parity-check (QC-MDPC) codes or binary Goppa codes [20]. SIKE, based on the Supersingular Isogeny Diffie–Hellman (SIDH) protocol, is not included in the scope of this paper since the SIKE team has acknowledged potential security issues.

Lattice-based cryptography algorithms rely on the hardness of the Shortest Vector Problem (SVP), which involves finding the shortest non-zero vector in a lattice and is known to be a difficult problem [21]. A lattice is a set of points in an *n*-dimensional space with a periodic structure defined by *n* linearly independent basis vectors [22]. The most commonly used problems in lattice-based cryptography are LWE and LWR. LWE involves finding the vector *s* given a matrix *A* and a vector b=As+e, where *e* is a small additive error [23]. If there is no error on the right side, the vector *s* can be easily found using Gaussian elimination. However, with a small additive error, this problem becomes very hard. Ring-LWE [24] and module-LWE [25] are the main variants of the LWE problem. Ring-LWE uses polynomial rings over a finite field as a domain, where the vectors *s* and *e* are polynomials from a polynomial ring [12]. On the other hand, module-LWE replaces the single-ring elements with a matrix of polynomials.

The hash-based algorithm was introduced by Lamport in the 1970s as a one-time signature scheme, where the key pair can only be used to sign one message [26]. However, this scheme is vulnerable if the same key is used to sign two different messages. To address this issue, the extended Merkle signature scheme (XMSS) was developed as a stateful signature that can be used multiple times [27]. Nonetheless, stateful signature algorithms encounter difficulty synchronizing states between different participants in communication [28]. SPHINCS+, on the other hand, is a stateless hash-based signature scheme that is a variant of XMSS but eliminates the need to maintain state.

Code-based cryptography was introduced by McEliece in 1978 [29]. This cryptosystem is based on the property of error-correcting codes that are easy to encode but hard to decode. The Classic McEliece uses the property of the Goppa code, which is a linear error-correcting code that can encrypt and decrypt a message [30] and is defined by a sequence of distinct *n* elements and a Goppa polynomial g(z). BIKE implements the McEliece scheme that uses QC-MDPC codes and the equivalent Niederreiter scheme. HQC employs a concatenated code of duplicated Reed–Muller and Reed–Solomon codes.

### 2.2. Core Operations for Post-Quantum Cryptography

The NIST PQC standards and round 4 candidates utilize the SHA-3 family [31] in their submissions, which comprises four hash functions and three extendable output functions (XOFs). A hash function takes input data of any size and generates a fixed-length digest, whereas an XOF produces variable-sized output data commonly used to randomly generate coefficients of polynomials in a lattice-based cryptosystem [13]. The hash functions in the SHA-3 family are based on the Keccak-*f* permutation, which is one of the most time-consuming components of PQC algorithms [12].

The Keccak-*f*[1600] permutation consists of iterative rounds that include five step mappings: θ, ρ, ϕ, χ, and ι, as shown in Algorithm 1. The input state for these mappings is an array of 25 elements, whose data size is determined by the permutation parameter and updated throughout the processes. The θ step mapping XORs all bits in two columns of the state array and XORs them with a bit-wise rotation. In the ρ mapping, the bits of the state array elements are rotated along the rotation offset, described as ROT in Algorithm 1, which depends on the *x*- and *y*-coordinates of the element. Then, the state array elements are rearranged in the ϕ step mapping. The bits in two state elements of different *x*-coordinates are XORed with a non-linear function in the χ mapping. Finally, the round constant, denoted as RC and depending on the current round index, is XORed with a state element, with both *x*- and *y*-coordinates being zero.
**Algorithm 1:** Keccak-*f* [1600] Permutation
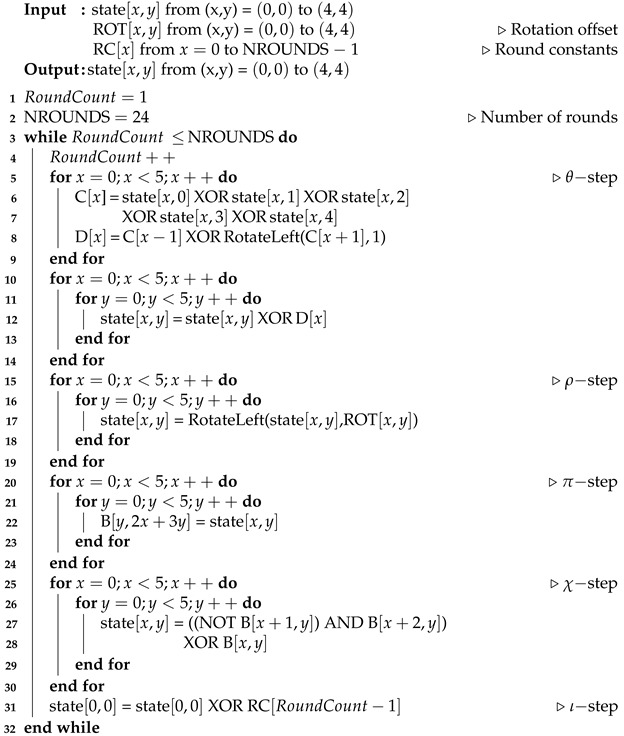


Polynomial multiplication requires high computation resources and is a time-consuming operation, so it is considered a bottleneck in lattice-based cryptography [32]. The naive method for polynomial multiplication, known as the schoolbook algorithm, has a computational complexity of $O(n^{2})$, where *n* is the degree of the polynomial. The number-theoretic transform (NTT) [33] is an efficient method for performing polynomial multiplication, as it can reduce the time complexity to O(nlogn).

(1) $${\hat{a}}_{i}=\sum_{j=0}^{n-1}a_{j}w_{n}^{ij}\;mod\;q,\forall i\in \;\left[0,\;n-1\right]$$

The NTT is a discrete Fourier transform in finite fields and is commonly used in post-quantum cryptography. It exploits polynomial multiplication over $R_{q}$, denoted by a polynomial ring $Z_{q}\left[x\right]/\left(x^{N}+1\right)$, where $Z_{q}$ represents the group of integers 0,⋯,q−1. $\omega_{n}$ is the *n*-th primitive root of unity in the finite ring $Z_{q}$, also known as the twiddle factor. The twiddle factor satisfies ${\omega_{n}}^{n}=1\;mod\;q$ and ${\omega_{n}}^{i}\neq 1\;mod\;q$ for all i∈[0,⋯,n−1].

(2) $$a_{i}=\frac{1}{n}\sum_{j=0}^{n-1}{\hat{a}}_{j}w_{n}^{-ij}\;mod\;q,\forall i\in \;\left[0,\;n-1\right]$$

The NTT and inverse NTT are defined as (1) and (2), respectively. Polynomial *f* contains the coefficient sequence $a=\{a_{0},\cdots ,a_{n-1}\}$ in $R_{q}$, whose degree is *n*. The polynomial *f* is transformed into $\hat{f}$ after the NTT, whose coefficient sequence is $\hat{a}=\left\{{\hat{a}}_{0},\cdots ,{\hat{a}}_{n-1}\right\}$. The NTT and inverse NTT can be described as $\hat{f}=NTT\left(f\right)$, $f=NTT^{-1}\left(\hat{f}\right)$.

(3) $$f\cdot g=NTT^{-1}\left(NTT\left(f\right)\circ NTT\left(g\right)\right)$$

**Figure 4:**
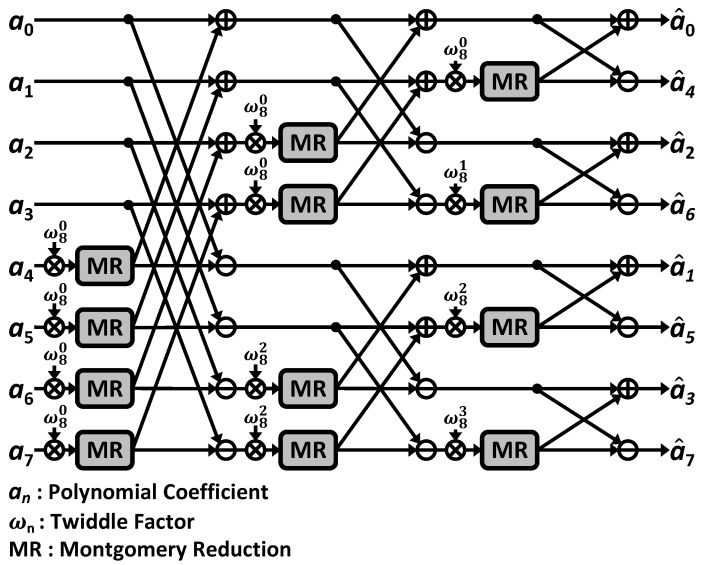
An 8-point CT butterfly-based NTT.

The NTT involves several computation stages that include butterfly operations. Figure 4 illustrates an 8-point NTT based on the Cooley–Tukey butterfly algorithm [33]. For an 8-point polynomial multiplication, three stages for each NTT and inverse NTT are required. The CT butterfly multiplies an operand with a twiddle factor, which is a power of a root of unity, and then begins addition and subtraction after modular reduction. In Kyber, Dilithium, and Falcon, Montgomery reduction [34] is utilized. Modular reduction is one of the main bottlenecks during polynomial operations [35]. Algorithm 2 describes how Montgomery reduction is performed on an integer *A*. The Montgomery factor is $\beta =2^{16}$ in Kyber and Falcon and $\beta =2^{32}$ in Dilithium.

Polynomial multiplication can be transformed into the component-wise multiplication of the NTT of polynomials *f* and *g*, denoted as ∘ in (3). NTT-based polynomial multiplication is achieved through iterative butterfly operations, which are detailed along with our proposed instruction set in Section 3. This property makes polynomial multiplication based on the NTT a target for accelerating efficient PQC computations.

Sampling coefficients for lattice-based cryptosystems is a performance bottleneck [16]. It is more efficient to sample coefficients from a centered binomial distribution than from a discrete Gaussian distribution [13]. The centered binomial distribution, which is symmetrical around zero, is generated by computing the Hamming weight of two η-bit values and then subtracting one from the other using a modular operation. Algorithm 3 describes the centered binomial distribution of width η. The parameter η determines the range of polynomial coefficients, which is [−η,η]. To generate uniformly random coefficients of polynomials that do not exceed a bound, rejection sampling can be used with the modulus *q* as the bound to generate the polynomial coefficients in $Z_{q}$.

The target code-based algorithms use arithmetic with bit polynomials over the finite field $GF_{2}[x]$, whose coefficients are either 0 or 1. Bit polynomial multiplication is a performance bottleneck in these algorithms [36,37]. In Classic McEliece and HQC, bit polynomial inversion over the finite field involves finite-field arithmetic, including repetitive multiplication and square operations. Accelerating the finite-field arithmetic can improve the execution time of the target code-based PQC algorithms.**Algorithm 2:** Montgomery Reduction for PQC algorithms
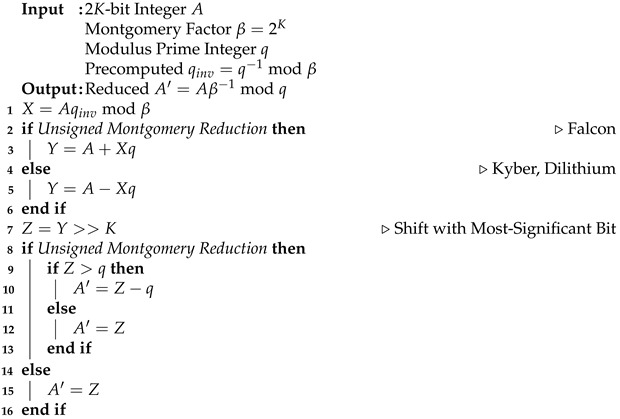

**Algorithm 3:** Sampling based on Centered Binomial Distribution
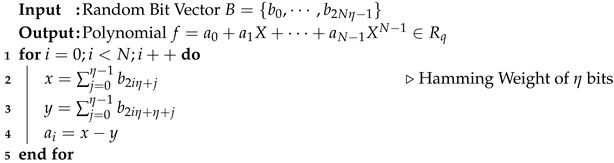


## 3. Proposed Crypto-Processor Architecture with PQC Instructions

To address the significant computational complexity and repetition involved in core operations in PQC algorithms, optimal hardware and software co-design is necessary. This section introduces the RISC-V instruction set extension that accelerates NIST PQC standards and round 4 candidates. Additionally, the hardware architecture of the proposed crypto-processor is discussed in detail.

### 3.1. Proposed RISC-V Instruction Set Extension

Due to its flexibility in instruction set extension, we use the RISC-V instruction set architecture as the baseline to support the NIST PQC algorithms and round 4 candidates [38]. As the primary performance bottlenecks in PQC algorithms are commonly due to the repetitive computation of similar core operations, a carefully designed instruction set extension can significantly enhance the efficiency of PQC computations.

The instruction format for the proposed RISC-V post-quantum cryptography extension is presented in Table 2. The basic formats in the RISC-V standard are Type-*R* and Type-R4. *R*-type instructions utilize two register values with indices *rs1* and *rs2*, whereas R4-type instructions require three indices for the source registers. In the proposed crypto-processor, each source register index in the R4-type instructions is used to access the 32 × 64-bit coprocessor register file (CR) instead of the general-purpose register in the baseline CPU core. Types Custom1 and Custom2 are newly defined PQC operations.

Table 3 provides a detailed description of the proposed instruction set, which can be classified into seven groups based on a comprehensive analysis of the target NIST PQC algorithms: Keccak-*f* permutation, Montgomery reduction for the NTT, binomial and rejection sampling, finite-field and conditional arithmetic, and access to coprocessor registers. Depending on the current instruction’s opcode, the source operands RS1 and RS2, as well as the destination register RD in Table 3, can be selected between the GPR in the baseline core and the CR. As shown in Table 4, the proposed crypto-processor supports all NIST PQC algorithms yet to be standardized and round 4 candidates.

In post-quantum cryptosystems, the Keccak-*f* permutation is the basis for SHA-3, encompassing hash functions (such as SHA3-256 and SHA3-512) and XOFs (such as SHAKE128 and SHAKE256). The number of iterations and the data size of the input and output array vary according to the permutation parameter, selectable from 25, 50, 100, 200, 400, 800, and 1600. The proposed instructions in this paper can accelerate any Keccak-*f* permutation parameter by simply adjusting the operand data size. For instance, for the Keccak-*f*[1600] permutation acceleration, the user can adjust the state array element data size to 64-bit and the number of rounds to 24. However, the Keccak-*f* permutation is time-consuming because of the high number of iterations, and it requires a significant number of registers for storing the permutation state. With the proposed PQC instructions, the five steps in Algorithm 1 for the Keccak-*f* permutation can be accelerated, as explained in Section 4.

As demonstrated in numerous prior works [14,32,39], accelerating the NTT can improve the performance of polynomial multiplication. Modular reduction is included in each operation in both the NTT and inverse NTT. Since the complexity of modular reduction is higher compared to other operations, such as addition, subtraction, and multiplication in the NTT, accelerating the reduction operation can efficiently improve the overall performance of the NTT. Montgomery reduction is the most commonly used reduction scheme in lattice-based cryptosystems that require polynomial multiplication. The proposed instruction set for Montgomery reduction supports all parameters used in PQC standards and round 4 candidates that use the NTT.

The parameter η in the binomial sampling instructions determines the number of bits used in the operation and the range of the result [−η,η]. For ease of use, Function 3 in the binomial sampling instruction set is equivalent to η, as shown in Table 3. The instruction set supports all possible values of η (2 and 3) used in NIST standard algorithms. In addition, the instruction set for rejection sampling includes REJH for sampling on 12-bit integers, which supports Kyber, and REJ for 23-bit integers, which supports Dilithium.

The finite-field arithmetic instructions consist of SQR for square operations and CLMUL and CLMULH for carry-less multiplication of bit polynomials. As discussed in Section 2, combining these fundamental operations can also speed up bit polynomial inversion.

CON4 and CON8 support conditional arithmetic operations. The result of the CON4 instruction depends on the value of RS1[15]. If RS1[15] is 0, the result is a 64-bit zero. If RS1[15] is 1, the result is RS2 as a 64-bit value. For CON8, the result of the instruction is determined by the value of RS1[31].

Recently, a scalar cryptography extension for RISC-V was announced, which supports the Advanced Encryption Standard (AES) and hash functions, including the SHA-2 family [40]. The proposed crypto-processor also includes a scalar cryptography extension to support NIST PQC standards, their variants, and finalists. Kyber introduced their 90s versions that use AES and SHA-2, and hardware acceleration of AES and SHA-2 can support SPHINCS+ [41]. AES is also used for random number generation in variants of PQC algorithms [42].

### 3.2. Proposed Crypto-Processor Microarchitecture

**Figure 5:**
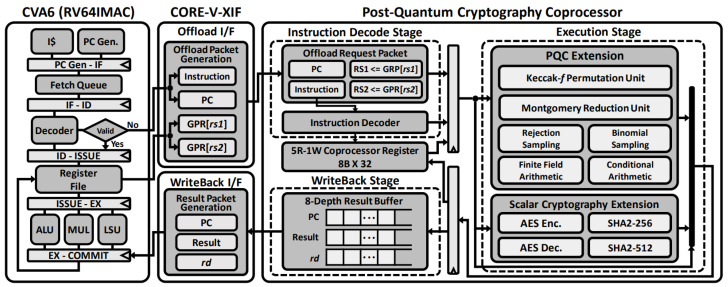
Overall architecture of the proposed crypto-processor.

Figure 5 illustrates the overall architecture of the proposed crypto-processor. The coprocessor interface, a subset of the CORE-V extension interface [15], connects the crypto-processor to the baseline CPU core, CVA6 [43]. CVA6 consists of six pipeline stages and supports the RV64IMAC ISA. When an instruction is fetched from the instruction cache, it is passed to the decode stage. Since CVA6 does not support the scalar cryptography extension or any proposed PQC instructions, they are considered invalid at the decode stage and offloaded through the coprocessor interface, with source operands from the general-purpose register in the baseline core, a 32-bit instruction, and the Program Counter (PC).

The crypto-processor has a three-stage pipeline architecture with an instruction decode stage, an execution stage, and a writeback stage, where all instructions require only a single cycle for the execution stage. The proposed coprocessor decodes the instruction with Function 7, Function 3, and Opcode fields to generate control signals. The Opcode determines the source operands for the current instruction from either the general-purpose register in the offload request packet or the coprocessor register. Based on the PC and source operands, as well as the index of the destination register and immediate value from the instruction, the execution stage of the coprocessor initiates an operation according to the control signals from the decode stage. The result is then passed to either the coprocessor register with the *rd* index or the general-purpose register through the writeback stage, along with the PC and index *rd*. The results that need to be written back to the baseline core are stored in the result buffer and wait to pass through the coprocessor interface. In the writeback interface, they become a result packet and are passed to the general-purpose register file in the commit stage of the baseline CPU core. Data stored in CR[*rs1*] can be loaded to the CPU core through the RDCR instruction, whereas the WRCR instruction allows a source operand in the general-purpose register to be written to the CR[*rd*]. The coprocessor register reduces frequent access to memory and the register file in the baseline core.

**Figure 6:**
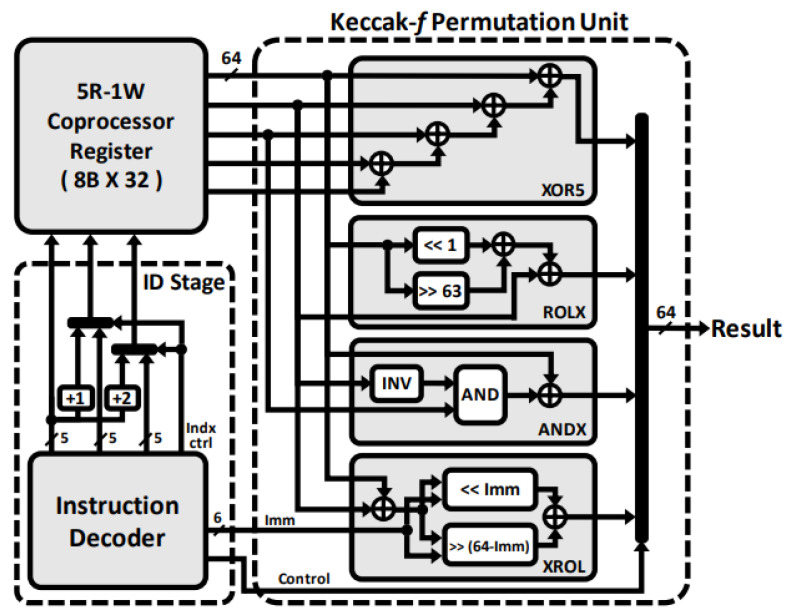
Hardware architecture of the Keccak-*f* permutation unit.

Figure 6 shows the hardware architecture of the proposed XOR5, ROLX, ANDX, and XROL instructions used in the Keccak-*f* permutation in the coprocessor execution stage. By utilizing a dedicated register file in the coprocessor to store the permutation state, repetitive access to the general-purpose register file in the baseline CPU core to update the state array every round can be avoided. Instead, these instruction sets utilize data stored in the coprocessor register as operands and write back computation results. During the decode stage, the index of the coprocessor register for each instruction’s source operand is determined.

The XOR5 and ROLX instructions support the θ step in the permutation. The XOR5 instruction accelerates repetitive 64-bit XOR operations with five 64-bit operands from CR[*rs1*] to CR[*rs1+4*], which would otherwise be executed for each clock cycle and repeated four times. The ROLX instruction first shifts the source operand CR[*rs1*] left by 1 bit and XORs it with CR[*rs2*]. The ANDX instruction supports the χ step in the Keccak-*f* permutation. In this instruction, the bit-inverted CR[*rs2*] and CR[*rs3*] are ANDed and then XORed with CR[*rs1*]. The XROL instruction accelerates the rest of the θ step and the whole part of the ρ and ϕ step mapping. The source elements CR[*rs1*] and CR[*rs2*] are XORed with each other and shifted by an amount specified in immediate[5:0]. The result is then written back to CR[*rd*].

**Figure 7:**
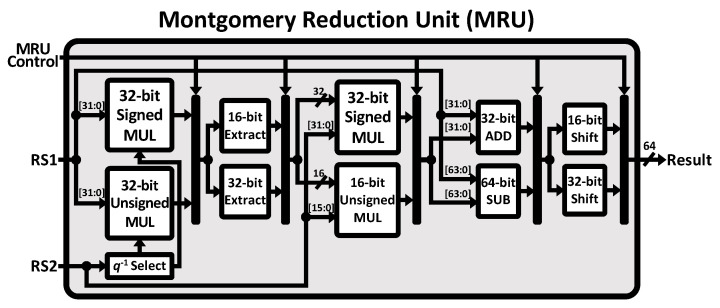
Hardware architecture of Montgomery reduction unit for the NTT instruction set.

The proposed Montgomery reduction instruction set is executed, as depicted in Figure 7. The parameter $q^{-1}$, which is the inversion of the prime factor *q*, is selected based on the value of RS2. The instruction decode stage controls the number of bits to be extracted and shifted and determines whether operands should be added or subtracted. MR4 and MR8 require 32-bit addition, whereas MR4U requires 64-bit subtraction. The shift amount is 16 bits for the execution of the MR4 and MR4U instructions and 32 bits for the MR8 instruction.

**Figure 8:**
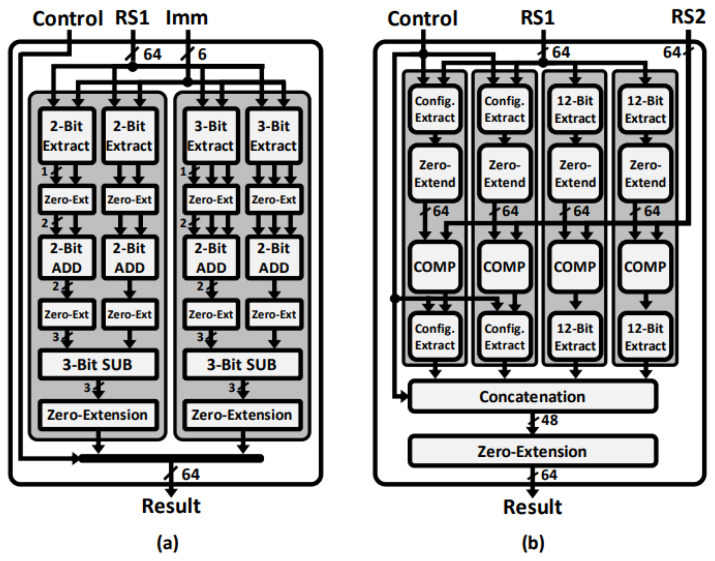
Hardware architecture of (**a**) binomial sampling unit; (**b**) rejection sampling unit.

Figure 8a shows the execution unit for sampling based on a centered binomial distribution. A 6-bit immediate determines the bit of the 64-bit source operand. Depending on the control signal from the instruction decode stage, η bits are extracted twice. The first set of η bits is obtained from RS1[Imm] to RS1[Imm+(η − 1)], and the second set of η bits is obtained from RS1[Imm+η] to RS1[Imm+(2η − 1)]. For instance, in the SND2 instruction, where η is 2, RS1[Imm] and RS1[Imm+1] are extracted, and each bit is extended to 2 bits by zero extension. Then the two 2-bit values are added, and the result is zero-extended to 3 bits. RS1[Imm+2] and RS1[Imm+3] are computed similarly. The two 3-bit values are subtracted, extending the result to 64 bits with zero. This produces the result of binomial sampling within the range of [−2,2]. The proposed instructions and crypto-processor support all η values used in the target NIST PQC algorithms.

The rejection sampling unit uses RS1 as the random bit stream generated by SHAKE or AES in PQC algorithms, and RS2 as the bound for rejection. By setting the bound as the modulus prime *q*, it is possible to select the polynomial coefficients within the appropriate range. The control signal from the instruction decode stage determines the bit widths of the configurable extractors, as shown in Figure 8b.

The execution stage also includes units for scalar cryptography [40]. The proposed crypto-processor can support AES encryption and decryption, as well as SHA-2 hash functions, including SHA-256 and SHA-512. These algorithms’ complex operations can be computed efficiently by a combination of appropriate instructions. For example, the AES unit supports instructions for key scheduling, middle encryption rounds (including ShiftRows, SubBytes, and MixColumns steps), and final encryption rounds (including ShiftRows and SubBytes steps). The instruction extension can also support AES decryption through inverse operations. For SHA-2 hash functions, transformations such as sigma0, sigma1, sum0, and sum1, which consist of rotation operations, are mapped to the RISC-V instruction format.

## 4. Experimental Results

This section presents a performance analysis of the proposed crypto-processor with PQC instructions. We evaluate the code size, number of executed instructions, and execution cycle counts. Furthermore, we compare the hardware implementation results with previous works.

### 4.1. Performance Analysis

In addition to reducing the cycle count, which was the primary focus of most previous works, the code size and number of executed instructions are also critical in demonstrating the effectiveness of the proposed instruction set. For the experimental setup in this study, we used a 64-bit CPU core, CVA6, as the baseline, and we employed the RISC-V GNU toolchain with the golden simulator, Spike. We measured the improvement based on the RV64IM with optimization level 3 (-O3) assembly source code of the target NIST PQC algorithms. As part of standardization, the NIST releases the C-based source code of each PQC algorithm, which we used in our experiments with the RISC-V compiler. To ensure a fair comparison, we modified only the assembly instructions that can be replaced with the proposed ISA and left everything else unchanged except for the target assembly instructions. We also provided execution clock cycle count reductions for each target cryptographic operation when the proposed crypto-processor was attached to the CVA6 core through the coprocessor interface, as shown in Figure 5.

Table 5 presents the comparison results for the Keccak-*f*[1600] permutation. The Keccak-*f*[1600] permutation cycle counts of the RV64IM software implementation based on the version 2.0 ISA for 32-bit [44], hardware–software co-design [45], and this work are shown in Table 5. Our study demonstrates that compared to the RV64IM software implementation of the permutation with optimization level 3, our proposed crypto-processor reduced the execution cycle counts by up to 86%.

Table 6 compares the NTT cycle counts according to the parameters used in NIST PQC standards. The parameter *n* refers to the degree of the polynomial, whereas *q* is a prime integer. Our work demonstrated a 46% to 50% reduction in cycle counts for the NTT compared to RV64IM with optimization level 3. Moreover, due to the well-defined instruction set, our proposed crypto-processor required fewer cycle counts for the NTT compared to previous works [14,32,39].

A comparison of execution cycle counts between the software implementation of sampling and previous works [13,46] is denoted in Table 7. The parameter *n* is the polynomial degree, and η determines the range of polynomial coefficients, as described in Section 2. As the rejection sampling in Kyber-512 reference implementation includes SHAKE-128, the proposed Keccak-*f* permutation and rejection sampling instruction set were used for the measurement. Our study shows that compared to RV64IM with optimization level 3, the proposed crypto-processor can reduce cycle counts for binomial sampling by 38% to 50% and rejection sampling by 77%.

Table 8 displays the execution clock cycle improvement for applications that use finite-field arithmetic, specifically the Syndrome decoder in mceliece348864 and the Reed–Solomon decoder in HQC-128. The reference implementation for NIST round 4 submissions was used for each experiment. The improvement of Karatsuba bit polynomial multiplication was measured based on the source code of the gf2x library [47], written in C. The code-based round 4 candidates, BIKE and HQC, use the gf2x library for efficient bit polynomial arithmetic, which can be supported by Karatsuba multiplication acceleration. $GF_{2}[x]$ polynomial multiplication significantly impacts the execution time of BIKE [36], and it is also utilized in all the primitives in the HQC implementation. The proposed crypto-processor exhibited execution cycle count improvements of 65%, 39%, and 87% for the respective algorithms.

**Figure 9:**
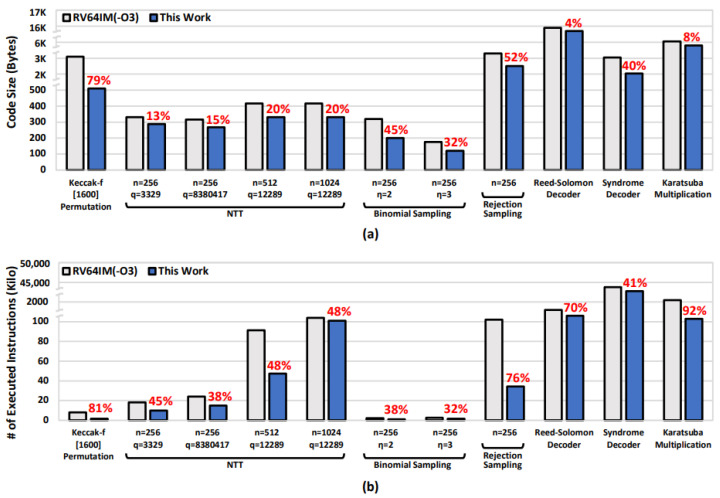
Comparison results of target applications. (**a**) Code size. (**b**) The number of executed instructions.

The reduction ratios for the code size and executed instructions are presented in Figure 9. For the Keccak-*f*[1600] permutation, the proposed instruction set achieved a reduction of 79% and 81% in terms of the code size and executed instructions, respectively. Regarding the NTT with standardized parameters for NIST PQC algorithms, the proposed instruction set achieved a reduction of 13% to 20% in terms of the code size and 38% to 48% in terms of the executed instructions. Additionally, the instruction set for binomial sampling achieved reductions of 32% to 45% and 32% to 38%, respectively. For rejection sampling, reductions of 52% and 76% were achieved in the corresponding experiments. Among the applications based on finite-field arithmetic, the proposed instruction set reduced the code size from 4% to 40% and the executed instructions from 41% to 92%.

### 4.2. Hardware Implementation Results

**Figure 10:**
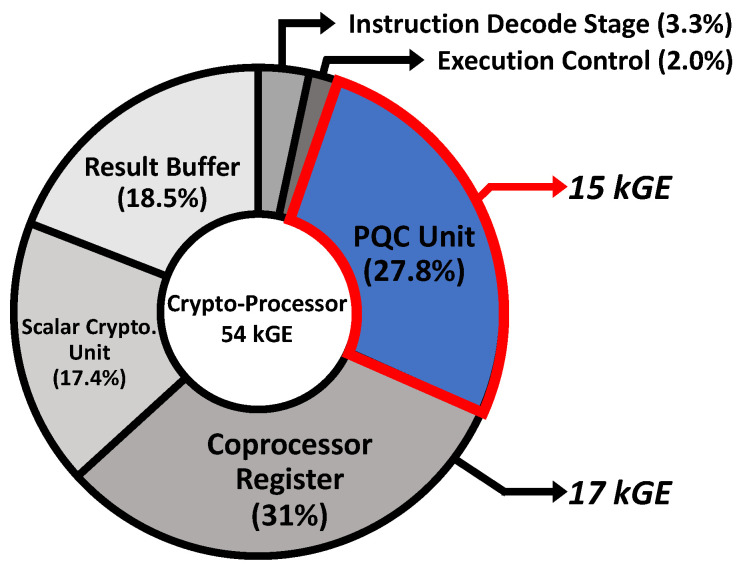
Gate-count ratio of proposed crypto-processor.

The proposed crypto-processor is described in Verilog HDL and synthesized with a 28 nm CMOS process at an operating frequency of 150 MHz. The total gate count is 54 kGE (NAND2 Gate Equivalent) and the ratio of each component is depicted in Figure 10. The PQC unit and scalar cryptography unit occupy 27.8% and 17.4% of the total gate counts, respectively. The 32 × 64-bit coprocessor register and the result buffer account for 31% and 18.5% of the coprocessor, respectively.

Although the gate count of the proposed crypto-processor is slightly higher compared to other tightly coupled PQC accelerators [13,14,32], as shown in Table 9, mainly due to the coprocessor’s register file and result buffer, which occupy about 50% of the total gate count, the proposed architecture as a programmable coprocessor supports all round 4 candidate algorithms in the NIST PQC standardization process, as well as algorithms yet to be standardized.

## 5. Conclusions

We propose an instruction set for all NIST PQC algorithms yet to be standardized and round 4 candidates, along with a programmable crypto-processor that can be easily attached to the baseline RISC-V CPU core as a coprocessor. This proposed architecture can alleviate computational bottlenecks in PQC algorithms, such as the Keccak-*f* permutation, the NTT, binomial and rejection sampling, and finite-field arithmetic. Additionally, the proposed crypto-processor supports RISC-V scalar cryptography extensions, including SHA-256, SHA-512, and AES. By combining the proposed instruction set with low complexity, the proposed architecture can support various PQC algorithms and cover various applications in real life. The proposed crypto-processor can reduce the code size and number of executed instructions of target operations in PQC algorithms by up to 79% and 92%, respectively. When compared to software implementations, execution cycle counts can be reduced by up to 87%. The proposed crypto-processor operates at 150 MHz and occupies 54 kGE with the 28 nm CMOS process.

Our proposed PQC coprocessor enables more secure communication compared to existing encryption systems. It is possible to keep information secure on communication channels with PQC algorithms. However, its disadvantage is that the operation of algorithms takes a long execution time. Our proposed crypto-processor can accelerate the computation of PQC algorithms to address these shortcomings. Therefore, the crypto-processor can be used in fields where PQC algorithms must be calculated in a short time by connecting to the main CPU.

The proposed ISA in this paper targets the PQC standard and round 4 candidate algorithms. It can also be applied to other algorithms, which can be accelerated by commands supported by the ISA. However, the limitation is that PQC algorithms that mainly use operations not supported by the proposed ISA cannot be accelerated. In this case, adding new instructions to the ISA will also reduce the computational time of various newly developed PQC algorithms, which is a potential avenue for future work in this paper.

## Figures and Tables

**Table 1:** NIST post-quantum cryptography standard and round 4 candidates.

Class	Algorithm Type	Public-Key Encryption/ Key-Establishment	Disigtal Signature
NIST PQC Standard	Latice	CRYSTALS-Kyber	CRYSTALS-Dilithium Falcon
	Hash	-	SPHINCS+
NIST PQC Round-4 Candidate	Code	BIKE HQC Classic McEliece	-
	Isogeny	SIKE	-

The SIKE teams acknowledged that SIKE is insecure and should not be used.

**Table 2:** Proposed crypto-processor’s 32-bit instruction format.

Type	Encoding Map																															
	**31**	**30**	**29**	**28**	**27**	**26**	**25**	**24**	**23**	**22**	**21**	**20**	**19**	**18**	**17**	**16**	**15**	**14**	**13**	**12**	**11**	**10**	**9**	**8**	**7**	**6**	**5**	**4**	**3**	**2**	**1**	**0**
*R*	Functon 7							*rs2*					*rs1*					Function 3			*rd*					Opcode						
*R4*	*rs3*					Func. 2		*rs2*					*rs1*					Function 3			*rd*					Opcode						
*Custom1*	*Immediate*							*rs2*					*rs1*					Function 3			*rd*					Opcode						
*Custom2*	Function 7							-					*rs1*					Function 3			*rd*					Opcode						

**Table 3:** The architecture of the proposed PQC instruction set.

Operation	Instruction	Description
Keccak-*f* Permutation	XOR5	RD = CR[*rs1*] ⌃CR[*rs1*+1] ⌃CR[*rs1*+2] ⌃CR[*rs1*+3] ⌃CR[*rs1*+4]
	ROLX	RD = ROL(RS1, 1) ⌃RS2
	ANDX	RD = CR[*rs1*] ⌃(∼CR[*rs2*] & CR[*rs3*])
	XROL	RD = ROL(RS1 ⌃RS2, *Imm*)
Montgomery Reduction	MR4	RD = (RS1 − ((RS1 × $q^{-1}$) & 64’hFFFF) ∗ RS2) >> 16
	MR4U	RD = (RS1 + ((RS1 × $q^{-1}$) & 64’hFFFF) ∗ RS2) >> 16
	MR8	RD = (RS1 − ((RS1 × $q^{-1}$) & 64’hFFFF_FFFF) ∗ RS2) >> 32
Binomial Sampling	SND2	RD = {61’b0, ({1’b0, ({1’b0, RS1[*Imm*]} + {1’b0, RS1[*Imm*+1]})}) − ({1’b0, ({1’b0, RS1[*Imm*+2]} + {1’b0, RS1[*Imm*+3]})})}
	SND3	RD = {61’b0, ({1’b0, ({1’b0, RS1[*Imm*]} + {1’b0, RS1[*Imm*+1]} + {1’b0, RS1[*Imm*+2]})}) − ({1’b0, ({1’b0, RS1[*Imm*+3]} + {1’b0, RS1[*Imm+4*]} + {1’b0, RS1[*Imm*+5]})})}
Rejection Sampling	REJH	RD[11:0] = (RS1[11:0] < RS2) ? RS1[11:0] : 12’b0 RD[23:12] = (RS1[23:12] < RS2) ? RS1[23:12] : 12’b0 RD[35:24] = (RS1[35:24] < RS2) ? RS1[35:24] : 12’b0 RD[47:36] = (RS1[47:36] < RS2) ? RS1[47:36] : 12’b0 RD[63:48] = 16’b0
	REJ	RD[23:0] = (RS1[22:0] < RS2) ? {1’b0, RS1[22:0]} : 24’b0 RD[47:24] = (RS1[46:24] < RS2) ? {1’b0, RS1[47:24]} : 24’b0 RD[63:48] = 16’b0
Finite Field Arithmetic	SQR	RD = {1’b0, RS1[31], 1’b0, RS1[30], ... , 1’b0, RS1[1], 1’b0, RS1[0]}
	CLMUL	RD = (RS2[0] ? (RS1 << 0) : 64’b0) ⌃... ⌃(RS2[63] ? (RS1 << 63) : 64’b0 )
	CLMULH	RD = (RS2[0] ? (RS1 >> 63) : 64’b0) ⌃... ⌃(RS2[63] ? (RS1 >> 0) : 64’b0 )
Conditional Arithmetic	CON4	RD = RS1[15] ? RS2 : 64’b0
	CON8	RD = RS1[31] ? RS2 : 64’b0
CR Access	RDCR	GRP[*rd*] = CR[*rs1*]
	WRCR	CR[*rd*] = GPR[*rs1*]

GPR: General Purpose Register in Baseline CPU Core. CR: Coprocessor Register. ROL: Rotate Left

**Table 4:** Supporting algorithms for the proposed PQC ISA.

Supportin Algorithm	Proposed RISC-V Post-Quantum Cryptography ISA					
	**Keccak-*f*** **Permutation**	**Montgomery** **Reduction**	**Sampling**	**Finite Field** **Arithmetic**	**Conditional** **Arithmetic**	**CR** **Access**
PKE/ KEM	Kyber	O	O		O	O
	BIKE	O		O		O
	HQC	O		O		O
	Classic McEliece	O		O		O
DS	Dilithium	O	O		O	O
	Falcon	O	O		O	O
	SPHINCS+	O				O

PKE/KEM: Public-Key Encryption/Key-Establishment, DS: Digital Signature

**Table 5:** Execution cycle counts (kilo cycles) for Keccak-*f*[1600] permutation.

RV64IM (-O3)	E31 (RV32IMAC) [44]	Cortex-M4 [44]	Accelerator [45]	This Work
11.722	13.774	12.969	1.8	1.632

**Table 6:** Execution cycle counts (kilo cycles) for the NTT.

Parameter (n, q)	Implementation				
	**RV64IM (-O3)**	**Accelerator [32]**	**Accelerator [14]**	**Cortex-M4F [39]**	**This Work**
(256, 3329)	25.53	43.76	18.49	-	13.88
(253, 8380417)	29.50	43.76	18.55	-	15.16
(512, 12289)	108.20	81.06		75.90	55.20
(1024, 12289)	237.74	180.24		157.70	119.98

**Table 7:** Execution cycle counts (kilo cycles) for sampling.

Target Algorithm	Parameter	Implementation		
		RV64IM (-O3)	Accelerator [13]	This Work
Binomial Sampling	n = 256, η = 2	2.46	-	1.24
	n = 256, η = 3	3.01	2.36	2.86
Rejection Sampling	Parameter	RV64IM (-O3)	Cortex-M4 [46]	This Work
	n = 256	206.36	60.43	47.36

**Table 8:** Execution cycle counts (kilo cycles) for applications based on finite-field arithmetic.

Target Algorithm	Implementation	
	**RV64IM (-O3)**	**This Work**
**Reed-Solomon Decoder (HQC-128)**	861.17	300.24
Syndrome Decoder (mceliece348864)	45,556.83	27,830.81
Karatsuba Multiplication	2311.75	305.98

**Table 9:** Comparison of hardware PQC accelerations.

Design	TCHE’20 [13]	IEEE Access’21 [14]	FPL’20 [32]	TCHE’19 [11]	TCAS-I’20 [16]	This Work
Platform	ASIC (65nm)	FPGA (ZCU106)	FPGA (VIRTEX-7)	ASIC (40nm)	ASIC (28nm)	ASIC (28nm)
Frequency (MHz)	45	100	-	72	300	150
Gate Counts (kGE)	$57^{\;a}$	-	-	106 ^*e*^	37 + $942^{\;f}$	477 + $54^{\;g}$
Complexity (LUT/FF/DSP/BRAM)	-	178/0/5/$0.5^{\;b}$ 377/0/10/$0.5^{\;c}$	417/462/0/$0^{\;d}$	-	-	-
Accelerator Type	Tightly Coupled	Tightly Coupled	Tightly Coupled	Memory-mapped	Coprocessor	Coprocessor
Supported NIST PQC algorithms	Kyber Saber	Kyber Dilithium	Kyber Dilithium Falcon	Kyber Dilithium	Kyber	Kyber Dilithium Falcon SPHINCS+ BIKE HQC Classic McEliece

^*a*^ Includes Pulpino w/o FPU. ^*b*^ Only for additional ALU logic for Kyber. ^*c*^ Only for additional ALU logic for Dilithium. ^*d*^ Only for additional accelerator. ^*e*^ Includes baseline CPU core. ^*f*^ SCR1 core and the vector coprocessor, respectively. ^*g*^ CVA6 core and the coprocessor, respectively

## Data Availability

The data in this study are available from the corresponding author upon reasonable request.
